# Designing an App for Parents and Caregivers to Promote Cognitive and Socioemotional Development and Well-being Among Children Aged 0 to 5 Years in Diverse Cultural Settings: Scientific Framework

**DOI:** 10.2196/38921

**Published:** 2023-02-13

**Authors:** Jacob J Crouse, Haley M LaMonica, Yun Ju Christine Song, Kelsie A Boulton, Cathrin Rohleder, Marilena M DeMayo, Chloe E Wilson, Victoria Loblay, Gabrielle Hindmarsh, Tina Stratigos, Michael Krausz, Nathanael Foo, Melissa Teo, Andrew Hunter, Adam J Guastella, Richard B Banati, Jakelin Troy, Ian B Hickie

**Affiliations:** 1 Youth Mental Health and Technology Team Brain and Mind Centre Faculty of Medicine and Health, University of Sydney Camperdown Australia; 2 Clinic for Autism and Neurodevelopment Research Brain and Mind Centre, Children’s Hospital Westmead Clinical School Faculty of Medicine and Health, University of Sydney Camperdown Australia; 3 Department of Psychiatry and Psychotherapy, Central Institute of Mental Health Medical Faculty Mannheim Heidelberg University Mannheim Germany; 4 Hotchkiss Brain Institute University of Calgary Calgary, AB Canada; 5 Department of Psychiatry Cumming School of Medicine University of Calgary Calgary, AB Canada; 6 Department of Radiology Cumming School of Medicine University of Calgary Calgary, AB Canada; 7 The Australian Prevention Partnership Centre Menzies Centre for Health Policy University of Sydney Sydney Australia; 8 Sydney School of Education and Social Work Faculty of Arts and Social Sciences University of Sydney Sydney Australia; 9 Department of Psychiatry University of British Columbia Vancouver, BC Canada; 10 Minderoo Foundation Perth Australia; 11 Australian Nuclear Science and Technology Organisation Sydney Australia; 12 Faculty of Arts and Social Sciences University of Sydney Sydney Australia

**Keywords:** early childhood development, digital technology, health information technology, mHealth, smartphone, neuroscience, pediatrics, mobile app

## Abstract

Recent years have seen remarkable progress in our scientific understanding of early childhood social, emotional, and cognitive development, as well as our capacity to widely disseminate health information by using digital technologies. Together, these scientific and technological advances offer exciting opportunities to deliver high-quality information about early childhood development (ECD) to parents and families globally, which may ultimately lead to greater knowledge and confidence among parents and better outcomes among children (particularly in lower- and middle-income countries). With these potential benefits in mind, we set out to design, develop, implement, and evaluate a new parenting app—Thrive by Five—that will be available in 30 countries. The app will provide caregivers and families with evidence-based and culturally appropriate information about ECD, accompanied by sets of collective actions that go beyond mere tips for parenting practices. Herein, we describe this ongoing global project and discuss the components of our scientific framework for developing and prototyping the app’s content. Specifically, we describe (1) 5 domains that are used to organize the content and goals of the app’s information and associated practices; (2) 5 neurobiological systems that are relevant to ECD and can be behaviorally targeted to potentially influence social, emotional, and cognitive development; (3) our anthropological and cultural framework for learning about local contexts and appreciating decolonization perspectives; and (4) our approach to tailoring the app’s content to local contexts, which involves collaboration with in-country partner organizations and local and international subject matter experts in ECD, education, medicine, psychology, and anthropology, among others. Finally, we provide examples of the content that was incorporated in Thrive by Five when it launched globally.

## Introduction

The first 5 years of human life are a remarkable period of cognitive, social, and emotional change. Before modern neuroimaging technologies, it was an open question as to how and to what extent the brain matures during childhood. Our understanding of this phenomenon is now much clearer, with a growing literature demonstrating large-scale structural and functional changes in the brain across childhood, starting from the very first months of life [1-6]. The development of the brain and its cognitive, social, and emotional functions during early life is critical for lifelong health and well-being. Levels of cognitive, social, and emotional functioning during childhood are associated with a variety of adult social, economic, and health outcomes [7-9], and children who struggle with some of these abilities (eg, self-control) when they are young are at elevated risk for negative outcomes as adults (eg, criminality) [9,10]. The degree to which child-rearing practices influence the development of these abilities during early childhood is of great interest.

Behavior genetics has demonstrated complex gene-environment interactions that, beyond a simple nature-nurture dichotomy [11,12], contribute greatly to how people differ in terms of cognitive, social, and emotional functioning. Notably, potentially modifiable environmental factors, such as what parents and families do with children, have a substantial influence on these differences. For example, a recent meta-analysis of twin studies estimated that around 40% of individual differences in self-control are attributable to environmental effects [13]. Twin studies have shown the contribution of the shared (family) environment to individual differences in language ability [14], empathy [15], and cognitive school readiness [16], among other traits. Epidemiologic studies have also revealed the need to protect children from harmful environments to ensure optimal cognitive, social, and emotional development [17,18]. Critically, exposure to certain harms (eg, chronic stress and abuse) might be avoided by educating parents and by equipping them and their children with protective strategies. Altogether, we now know that childhood cognitive, social, and emotional traits are malleable. Improving children’s functioning in early life via parental behaviors may optimize development and prevent poor outcomes in adulthood, ultimately resulting in lifelong health and well-being.

Modern digital technologies (eg, smartphone apps) offer a highly scalable platform for delivering health information across a range of settings (eg, low- and middle-income countries), purposes (eg, education and prevention), and health conditions [19-22]. In an increasingly web-based world, app-based technologies offer the potential to address ethnic, racial, socioeconomic, and regional disparities in access to health information and may represent an effective means of delivering information about early childhood development (ECD) to parents and caregivers globally. Realizing these opportunities hinges on investigating and interrogating the social, cultural, and political dimensions of digital technology use [23], as well as conducting detailed examinations of how users of digital technologies perceive a specific platform’s usability (eg, user-friendliness), acceptability (eg, cultural appropriateness), and feasibility (eg, ease of use in daily life) [24].

**Figure 1:**
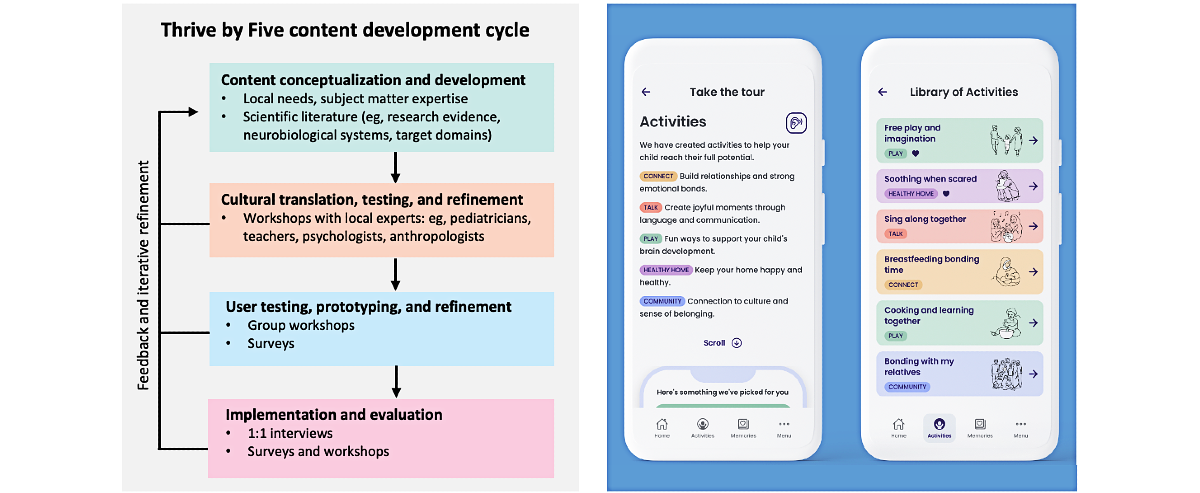
Overview of our scientific framework for developing the Thrive by Five app. We combine cultural and anthropological analyses and scientific knowledge to develop and iterate country-specific practices for parents and significant others (collective actions).

With the goal of meeting these challenges and opportunities, we partnered with a philanthropic organization—Minderoo Foundation—in 2021 to develop, implement, and evaluate an app that aims to provide parents from approximately 30 countries with science-based and culturally relevant information about ECD. At the time of writing, the app—Thrive by Five—has been launched as 5 localized, country-specific versions. A key difference between Thrive by Five and other popular parenting apps is our focus on combining scientific knowledge with a cultural and anthropologic analysis of each country’s local context (eg, approaches to child-rearing, gender roles, and the position of the child in the family; Figure 1). Moreover, rather than focusing only on 1 parent and 1 child, we broadened the scope of the app’s child-rearing tips to draw in wider family and community networks, with the goal of exposing children to a wider set of cultural and traditional practices (eg, folk stories; myths; and traditional songs, music, and dance). Accordingly, we refer to the activities in the app as *collective actions*.

The objective of this paper is to describe this project’s scientific framework. By *scientific framework*, we refer to the project’s basic conceptual and pragmatic approach, including *what* we are targeting (ie, cognitive, social, and emotional well-being); *why* these targets are of interest (ie, the empirical, scientific rationale); and, critically, *how* users can engage with specific practices to potentially drive their children’s development in these target areas in ways that are culturally relevant. As our approach to integrating science, culture, and anthropology within a co-design context is novel, the rationale of describing our scientific framework is to provide a road map that other projects with similar aspirations may find useful, as well as a transparent description of the process underlying the design and development of the Thrive by Five app.

## Methods

### Scientific Framework Overview

In the following sections, we elaborate on this project’s scientific framework. First, we describe 5 conceptual domains that are used to organize the content and goals of the information about ECD and child-rearing and outline 5 neurobiological systems that can be behaviorally targeted to influence social, emotional, and cognitive development (*Scientific Framework Part 1*). Second, we discuss our approach to developing an understanding of each country through the cocreation of a cultural framework that summarizes various literature regarding factors that may impact child-rearing and child development (*Scientific Framework Part 2*). Third, we introduce the concept of collective actions as an alternative to parenting tips, emphasizing the strengths of involving wider family and community networks in child-rearing (*Scientific Framework Part 3*). Fourth, we discuss our iterative approach to localizing the app’s content for each country, which involves holding collaborative workshops with in-country partner organizations; subject matter experts in ECD, education, medicine, psychology, and anthropology, among other disciplines; and potential users of the app in each country (*Scientific Framework Part 4*). Finally, we provide examples of the content (ie, collective actions) that was included in the app when it launched internationally in 2022 (the first full version of the app was implemented in Indonesia).

#### Scientific Framework Part 1—Linking Content Development to 5 Thematic Domains and 5 Neurobiological Systems

**Figure 2:**
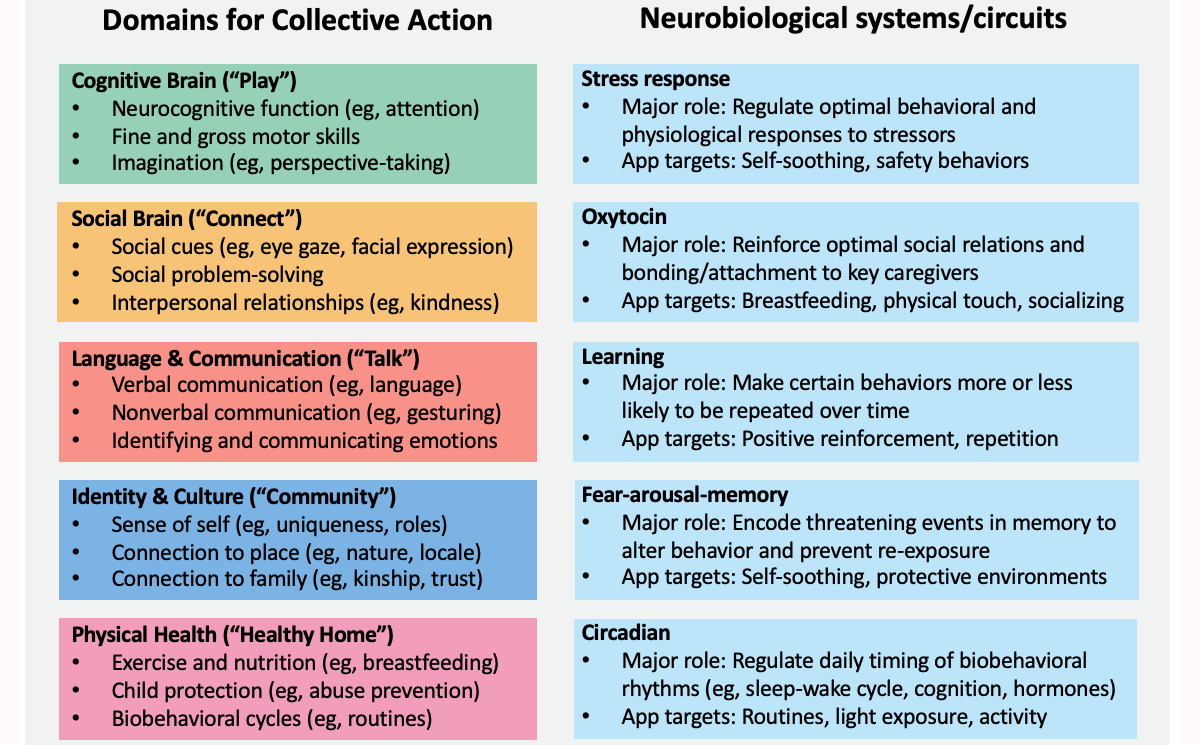
The five domains for collective action and the five neurobiological systems/circuits used to guide the conceptualization and development of the app’s content.

Before developing the app’s content, we agreed on 5 thematic domains that are relevant to children’s social, emotional, and cognitive development and 5 neurobiological systems that are involved in social, emotional, and cognitive development. These domains and neurobiological systems (Figure 2) guide the development of the app’s content, are used to categorize the content within the app, and provide the scientific rationale for encouraging parents to engage with the practices promoted by the app.

The thematic domains are based broadly on the Bright Tomorrows project (developed by Minderoo Foundation and Telethon Kids Institute), with the Brain and Mind Centre team mapping new domains. The domains and the broad types of content included in each domain are (1) the *Cognitive Brain* domain, which includes content about broad cognitive processes (eg, attention, learning, memory, visual and auditory processing, motor skills, and imagination); (2) the *Social Brain* domain, which includes content about social interaction and the sociocognitive processes involved in recognizing, interpreting, and responding to social cues (eg, eye gaze, joint attention, and facial expressions); (3) the *Language and Communication* domain, which includes content about processing, understanding, and using verbal and nonverbal language and signals (eg, gestures); (4) the *Identity and Culture* domain, which includes content about the development of a sense of personal, social, and community identity and the roles that culture and place play in identity development (eg, customs, festivals, and folk stories); and (5) the *Physical Health* domain, which includes content about physical health, growth, and development and physical protection from harm and abuse (eg, harsh discipline).

The neurobiological systems that we focus on and an outline of their relevance to early child development are shown in Figure 2. These five systems and their main functions include (1) the stress response system, which creates a hormonal response to stress (prolonged activation of the stress response system is associated with negative emotional, behavioral, and physical health outcomes); (2) the oxytocin system, which regulates social, behavioral, and emotional processes (eg, smiling, attention to eye gaze, and breastfeeding), of which many are fundamentally important for early child-caregiver bonds and other social bonds; (3) the learning system, which assigns value to objects and behaviors (in childhood, this is fundamental for motivation creation, social behaviors, and associative learning); (4) the fear-arousal-memory system, which encodes and maintains memories of fearful stimuli and the contexts in which they are experienced; and (5) the circadian system, which orchestrates the daily rhythmic timing of almost all physiological processes and behaviors (eg, sleep and wakefulness, appetite, mood, and cognitive function). Other publications provide more details about these systems and their relevance to early child development [25-38].

#### Scientific Framework Part 2—Development of Cultural Frameworks to Localize Content

**Figure 3:**
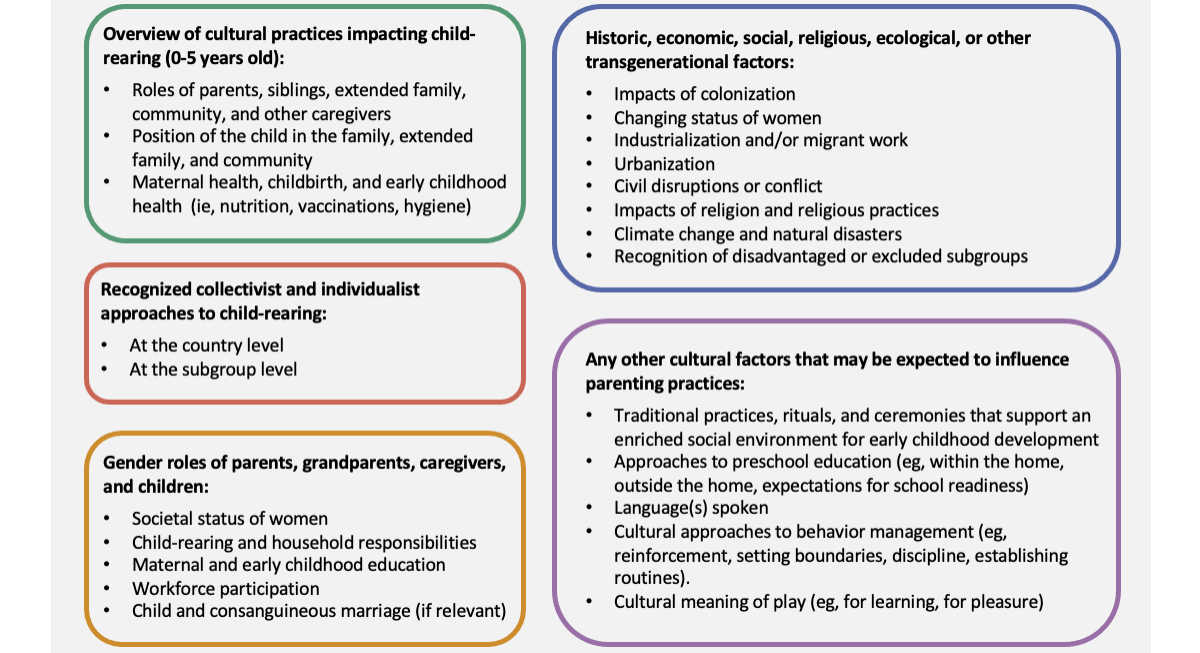
Components of the cultural framework prepared for each country.

For each country, a cultural framework is developed collaboratively with the research team and a nominated country-specific expert. This framework is used to guide the first draft of the app’s content for each local context. The cultural framework summarizes information from a variety of published literature (eg, government reports, journal articles, and textbooks) that is relevant to child-rearing; family environments; and broader social, economic, and political factors that may influence family functioning and early child development. For each country, we follow a dedicated pro forma that covers the topics presented in Figure 3.

Concurrently, the research team prepares a literature summary that presents the strengths (eg, transgenerational family networks; the empowerment of women; and the cultural celebration of art, music, and dance) and challenges (eg, high rates of childhood mortality, obesity, and exposure to corporal punishment) of each country, which are considered when developing the app’s content. The content aims to celebrate the cultural strengths and practices of each country by highlighting how they align with the scientific evidence about childhood development while also considering the various challenges that may impede these practices and how these challenges may be mitigated. Once complete, the cultural framework and literature summary are reviewed and approved by an in-country partner organization.

#### Scientific Framework Part 3—Conceptualizing the App’s Content as Collective Actions and Not Just as Parenting Tips

Many available parenting apps adopt a narrow focus in their content, encouraging activities to be completed by 1 parent (typically a mother) with 1 child. Although these activities are well-meaning and are still of potential benefit, their dyadic structure is limiting. Such activities reduce exposure to a variety of social interactions with different people; lack the richness of multigenerational and extended family structures; and narrow the bounds of the complexity and variety of children’s social, emotional, and cognitive experiences. Therefore, we conceptualize the content in the app not as parenting tips but as collective actions.

**Figure 4:**
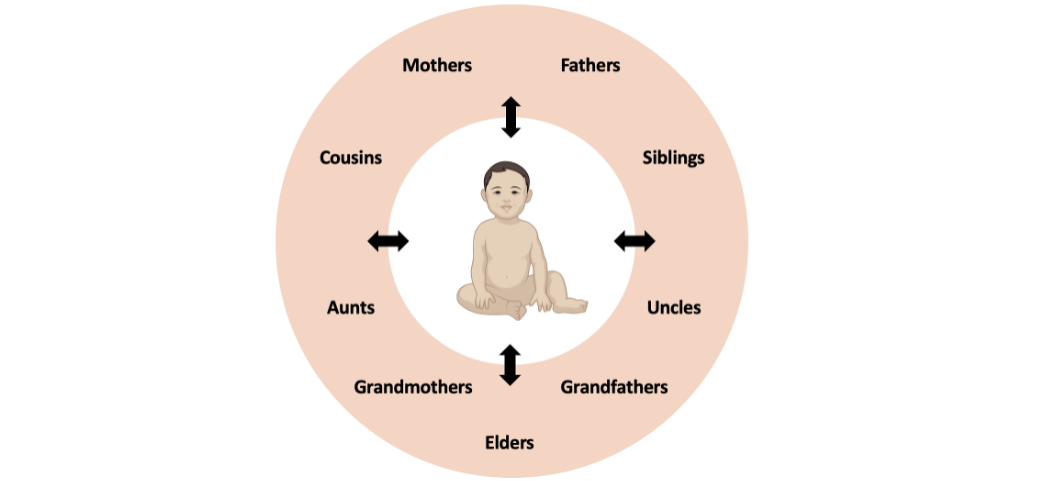
Our approach to content: collective actions, not just parenting tips. As opposed to a set of child-rearing practices that encourage simplistic parent-child interactions, our concept of collective actions encourages involvement from many family members (eg, grandparents, siblings, and cousins) and trusted community members in interactions with the child. Although we have placed the child at the center of this network, we also recognize the child as an “actor,” as children often initiate interactions with others, and that these communications represent interactive loops rather than a unidirectional interaction.

These collective actions broaden the scope of the child-focused activities to include a significantly larger network of individuals in a child’s life. As well as mothers, fathers, uncles, aunts, siblings, cousins, grandmothers, and grandfathers (Figure 4), we also encourage families to bring in other trusted adults from their communities and social networks to engage in these actions. We believe that this wider circle of interactions with and around a child may drive greater gains in cognitive, social, and emotional development (by increasing the varieties of experiences, stimuli, and interactions), in addition to increasing the opportunities for a child to be exposed to and embedded within the rich tapestries of their extended family’s culture and history (eg, rituals; folk stories; myths; and traditional songs, music, and dance).

**Figure 5:**
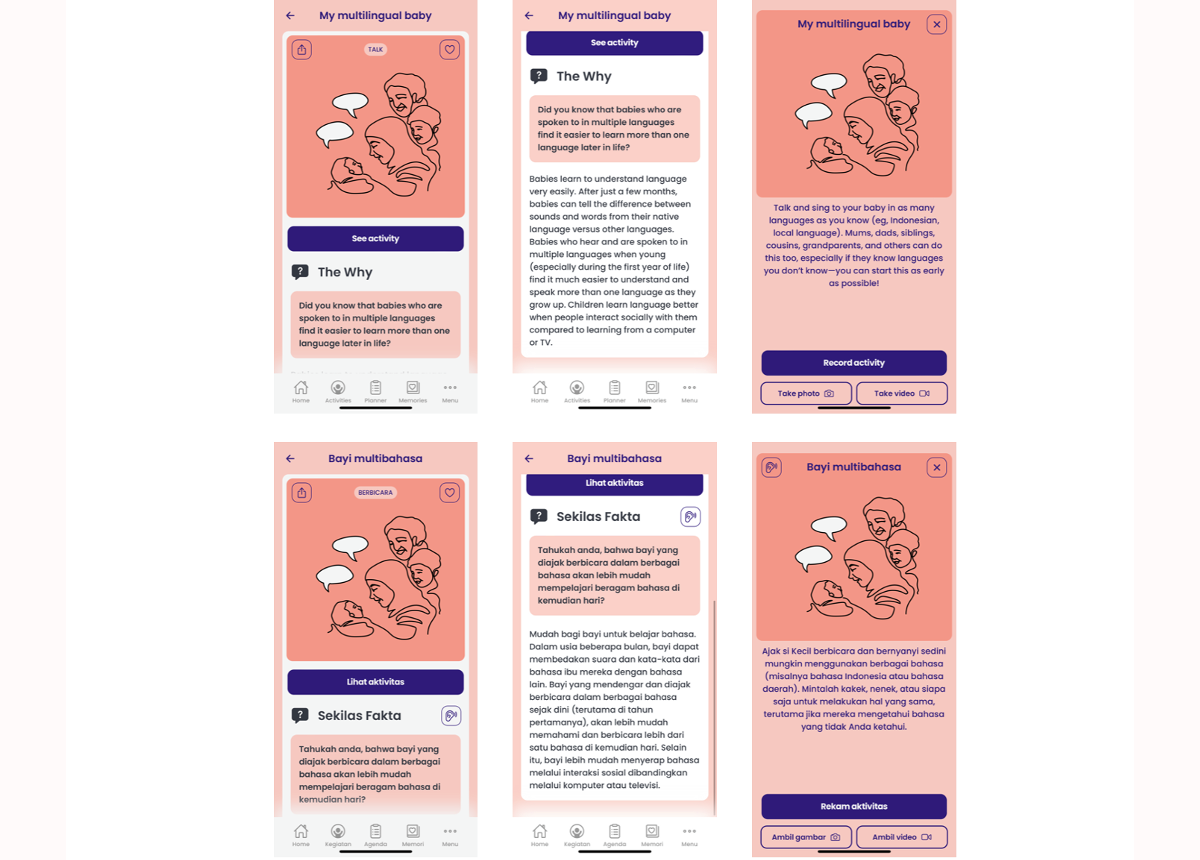
An example of a collective action, including “The Why” and “Activity Pop Up” components in English (top row) and Bahasa Indonesia (bottom row).

In the Thrive by Five app, the primary content (collective actions) comprises 2 components (Figure 5). First, “The Why” provides the scientific background that supports a particular activity (eg, the importance of breastfeeding for a baby’s social, cognitive, emotional, and physical development and health outcomes) [39,40]. Second, the “Activity Pop Up” provides a practical activity in which parents, siblings, grandparents, other extended family members, and trusted community members could participate with the child. “The Why” and the “Activity Pop Up” are available both in text form and in audio form.

#### Scientific Framework Part 4—The Iterative Process of Localizing Content to Each Country

Over the course of this 3-year project, the Thrive by Five app will be implemented in 30 countries. A unique feature of this project is that for each country, we are actively considering how we can integrate cultural practices into the collective actions as a way of strengthening connections across families and communities and connections to places, cultural traditions, and values. To ensure that the app’s content is culturally acceptable, usable, relevant, and engaging, we developed a 4-stage process for developing and prototyping the country-specific collective actions (Figure 1) in partnership with local and international experts in ethnography, anthropology, ECD, medicine, psychology, and other disciplines.

The first phase of development is the conceptualization of the initial library of collective actions for a given country. This process is guided by the research team’s expertise and an examination of published research in areas that are relevant to the five domains and the five neurobiological systems that we previously described (Figure 2). The cultural framework (Figure 3) is used concurrently to highlight topics regarding local needs (eg, hygiene and distress management).

In the second phase of development and prototyping, the research team—in cooperation with a nominated in-country partner and representatives from the Minderoo Foundation—holds a series of co-design workshops with local subject matter experts (eg, educators, pediatricians, and psychologists) to examine the acceptability, feasibility, and relevance of the draft content (more details are provided elsewhere [41]). Similarly, in the third phase of development and prototyping, the research team holds a series of workshops with in-country parents, using a beta version or clickable demonstration version of the app to further examine the acceptability, usability, and relevance of the content. Based on the data that emerge from the co-design workshops, the drafted content is iteratively revised.

Finally, the last phase of development and prototyping includes the implementation of the app in the given country, after which an evaluation phase is conducted that examines the impacts of the Thrive by Five app on several factors, including parent-level confidence and self-efficacy (more details are provided elsewhere [41]). Importantly, at any one of the phases that involve communication with in-country partners, experts, and parents, ideas for subsequent collective actions may emerge.

## Results and Discussion

The international launch of the Thrive by Five app was in March 2022, marked by the implementation of the first full version of the app in Indonesia. Further, 4 other versions of the app (with localized country-specific content) have been successfully implemented in Afghanistan, Namibia, Kyrgyzstan, and Uzbekistan as of November 2022, and 5 other country-specific libraries of localized content have already been codeveloped and are awaiting implementation. As this project continues to progress, the team will codevelop 20 country-specific libraries and implement the app in 25 countries (bringing the total to 30 countries).

A series of evaluation studies will investigate the parent-level, family-level, and system-level impacts of the app on several factors, including the perceived connection between parents and children and between children and the community, parents’ confidence in their caregiving abilities, and knowledge gain with regard to positive child-rearing practices [41]. These evaluations will include a mix of quantitative and qualitative designs and a mix of country-specific investigations and larger cross-country investigations. We anticipate the first empirical reports from this program to be submitted for publication in early 2023.

We acknowledge several limitations of our approach. First, while we are co-designing each of the collective actions (collaborating with potential users and local experts) and the aspects of the app’s design (eg, illustrations) [41], several of the features and functions of the app were developed before the co-design phase. Second, in some instances, we have had strong feedback from co-design workshop participants about the relative lack of content related to religious practices. Although obviously culturally relevant, we decided that many of these suggestions would not be included in the app’s content, as we could not be confident about their relationships with the cognitive, emotional, and social outcomes of this project. Third, we recognize that while some of our team members have personal experiences with the countries for which we are developing the app, we cannot truly understand the nuances, particularities, and meanings of each country’s cultural practices (A Poulsen et al, unpublished data, 2022) [42]. In these instances, we adopt a position of cultural humility and rely more heavily (and modestly) on collaboration with our in-country partners and workshop participants. We hope that we can learn and understand enough to make each app feel authentic and relevant to the users.

Our approach to developing the Thrive by Five app—combining science with cultural and anthropological knowledge—is highly original, and we hope that it will produce a useful, relevant, and engaging resource for parents and families around the world. The first outcomes from this work are expected to be published in early 2023. In closing, this global project brings together cutting-edge knowledge from neuroscience, ECD, digital technology, and anthropology, with the major goal of empowering families around the world with new tools and practices for shaping their children’s futures.

## Abbreviations

ECD: early childhood development
